# Gendered socio-economic and mental health effects of the COVID-19 pandemic among adults living in selected informal settlements in Kenya: an intersectional analysis

**DOI:** 10.3389/fpubh.2024.1469091

**Published:** 2024-10-29

**Authors:** Daniel M. Mwanga, Henry Owoko Odero, Damazo T. Kadengye, Sally Atieno Odunga, Eva Muluve, Bylhah Mugotitsa, Ruth Nanjekho Wafubwa, Karen Austrian, Sylvia Kiwuwa-Muyingo

**Affiliations:** ^1^African Population and Health Research Center, Nairobi, Kenya; ^2^Department of Mathematics, University of Nairobi, Nairobi, Kenya; ^3^Department of Economics and Statistics, Kabale University, Kabale, Uganda; ^4^Population Council, Nairobi, Kenya

**Keywords:** mental health, COVID-19, income loss, food insecurity, access to health services, gender intersectionality

## Abstract

**Background:**

COVID-19 pandemic had devastating socio-economic and health effects, including mental health. This study examines the intersectionality between gender and mental health outcomes among Kenyan adults in informal settlements of Nairobi, Kisumu, and Kilifi Counties during the COVID-19 crisis. This is necessary to inform mental health response in case of another pandemic.

**Methods:**

We analyzed data collected in a longitudinal survey between July 2020 (fourth round) and February 2021 (fifth round). The data covered COVID-19-related effects on job loss, food insecurity, access to health services, and mental health. Participants were randomly sampled from existing cohorts at the Population Council. The outcomes of interest were depressive and anxiety disorders, combined into a binary composite outcome variable. Descriptive statistics included means for continuous variables and frequencies and proportions for categorical variables. Chi-square tests were used to examine the differences between groups. The relationship between the gendered COVID-19 socio-economic effects and participants’ mental health was examined using modified Poisson regression.

**Results:**

A total of 4,050 participants were interviewed, 66% female and median age 38 [interquartile range (IQR): 29–46]. Complete income loss was strongly associated with negative mental health outcomes in multiple intersections with varied magnitudes. The association was larger among older females (≥50 years) (PR = 1.33, 95% CI = 1.17–1.51, *p* < 0.001) than older males (PR = 1.22, 95% CI = 1.17–1.27, *p* < 0.001). Partial loss of income was protective against negative mental health outcomes among young males (18–29 years) (PR = 0.81, 95% CI = 0.76–0.87, *p* < 0.001) but linked to negative outcomes among middle-aged males (30–49 years old) (PR = 1.14, 95% CI = 1.12–1.16, *p* < 0.001). Skipping meals was associated with negative mental health outcomes for both genders particularly the married (married male: PR = 1.49, 95% CI = 1.22–1.83, *p* < 0.001; married female: PR = 1.42, 95% CI = 1.26–1.60, *p* < 0.001).

**Conclusion:**

We observed significant gender differences in the prevalence of depressive symptoms and anxiety disorders during the COVID-19 pandemic, underscoring the importance of socio-economic factors and health services access in shaping mental health outcomes. Interventions targeting pandemic-related mental health issues should be gender-sensitive and address economic vulnerabilities such as job losses and food insecurity. Policies to mitigate these effects, especially for at-risk groups are crucial for reducing mental health burden in future crises.

## Background

The COVID-19 pandemic is one of the most significant global crises of the 21st century, which affected various aspects of human life across the globe (1–5). The World Health Organization (WHO) declared it a global pandemic with 351,500 confirmed cases and 22,300 deaths globally as of March 29, 2020 (6). To contain the pandemic, several national governments worldwide implemented various containment measures such as lockdowns, wearing of face masks, quarantines, travel restrictions, and social distancing guidelines, to contain the spread of the disease (6). Consequently, socio-economic amenities including retail and commercial businesses, social places, and schools were disrupted to reduce transmission rates within communities. While these containment measures slowed down the spread of the virus, they resulted in the loss of employment, disrupted daily schedules and social networks for many people and economic uncertainty became widespread resulting in financial instability and food insecurity among several households globally (7).

The different containment measures unintentionally increased social isolation and loneliness, especially for vulnerable populations like the older adults (8) and those living in informal settlements. Consequently, individuals experienced heightened levels of anxiety, depression, and related mental health issues (9, 10). These effects are more pronounced in low- and middle-income countries (LMIC) especially in sub-Saharan Africa (SSA) (11, 12). World Bank statistics show that on average, 30% of households in SSA countries experienced job losses with Kenya’s highest at 62%. Further, statistics show that 1 in 3 household enterprises in Kenya, Nigeria, and Ethiopia closed at the outset of the pandemic (13).

Existing evidence suggests that the prevalence, presentation, and pathways of mental health conditions are significantly influenced by a person’s gender (14–16). Studies from previous pandemics have shown that during emergencies, women often face heightened vulnerabilities such as disproportionate income losses and negative health outcomes because of instituted policy measures. Similar consequences of restrictive policy measures have been previously experienced during the 1918–1919 Influenza Pandemic (17), the 2013–2016 Ebola virus epidemic in West Africa (18, 19), and the 2016 public health emergency of the Zika virus in Southern America (20). Economic consequences of restriction measures amplify pre-existing inequalities along factors such as gender, age, and geographical location.

Several studies have been conducted on COVID-19 in informal settlements in Kenya including on COVID-19 vaccine hesitancy (21, 22), socio-economic impacts of the pandemic (4, 23), and knowledge, attitudes, and practices (24). Most of these studies have focused on the social and economic effects of the pandemic containment measures while studies on mental health have focused mostly on adolescents (5) and the mental health outcomes at aggregate levels (25). While there is growing evidence of the disproportionate impact of COVID-19 on different demographic groups, there remains a notable research gap on the gendered relationship between the effects of COVID-19 containment measures on mental health with a specific focus on vulnerable African contexts (26, 27) under different intersections between gender and demographic subgroups such as age, residence, and marital status. It is necessary to use the data from the COVID-19 pandemic to inform response strategies to reduce mental health effects in the event of another pandemic. In this paper, we examine the impact of COVID-19 containment measures on the population mental health of adult men and women in informal settlements. We explore how the different socio-demographic characteristics intersect with each other resulting in deferential mental health outcomes among men and women in Kenya.

## Materials and methods

### Study population and the survey design

We analyzed data collected by the Population Council Kenya among five informal settlements of Nairobi, two peri-urban settlements of Kisumu, and rural Kilifi in July 2020 and February 2021. The study was designed as a longitudinal survey to monitor how the study communities were affected as the COVID-19 pandemic progressed. Detailed survey methods, design, description of the population and why they were selected in the original cohort from where the sampling was done, sampling procedures, sample sizes, and inclusion and exclusion have been described elsewhere (5). In brief, participants were identified and sampled from existing studies in a ratio of 1:3 for male-to-female interviews. Women were oversampled due to interest in sexual and reproductive health outcomes. Remote surveys were used during COVID-19 lockdowns and mobile phone interventions were employed to collect data. The surveys were designed to provide rapid information to inform the COVID-19 response in informal settlements. As of February 2021, there had been five rounds of data collection in Nairobi and two rounds in Kisumu and Kilifi with the latter two being introduced in the fourth round. In Nairobi, the first round was conducted in March 2020, the second round in April 2020, the third round in May 2020, the fourth round in July 2020, and the fifth round in February 2021. A module on mental health on module introduced in the fourth round and expanded the sample to include Kilifi and Kisumu. This study therefore utilizes data only from the fourth and the fifth rounds. All participants who responded to the survey in rounds 4 and 5 were included in the analysis. The specific mental health outcomes captured in the tool were depressive symptoms, using the standard patient health questionnaire tool (PHQ-2), and anxiety disorders (introduced in the fifth round) using the standard generalized anxiety disorder scale (GAD-2). The simpler versions of PHQ and GAD were chosen because the study was designed as a rapid survey and the focus was on getting quick data to inform the COVID-19 response in Kenya’s informal settlements.

Data collection was conducted over the phone utilizing a database of existing studies in the study locations because of COVID-19 containment measures that restricted person-to-person interactions (5). During the pandemic, the government put in place containment measures including lockdowns in high-risk areas, and restricting physical person-to-person interactions for non-essential services. This meant that the only way to collect data was to utilize mobile phone surveys where participants whose contacts existed in the databases created by the studies documented in (5) were called and the questionnaire administered. The survey questionnaire took approximately 45 min to complete covering topics such as COVID-19 knowledge, attitudes, and practices including myths and misconceptions to inform COVID-19 messaging. Findings on these have been published (24).

All survey instruments in English, Swahili, and Luo languages, were designed and programmed in Open Data Kit (ODK). Data collection was conducted over the phone and submitted to an online server. All data were cleaned and exported to Stata version 17.1 for analysis.

### Ethical considerations

Ethical approval was obtained from both the Population Council Institutional Review Board (p936) and AMREF ESRC (P803/2020). The study also acquired a research permit from the National Commission for Science, Technology, and Innovation (P/22/16531). Participants who were 18 years and older provided verbal consent while those under 18 years provided verbal assent in addition to their parents’ or guardians’ consent after the informed consent was read to them. Participants were reimbursed 100 Kenyan Shillings (∼US$1) to compensate them for their time spent on the survey. Reimbursements were sent via MPESA mobile money transfer.

## Variables

### Primary outcomes

#### Depressive symptoms

Depressive symptoms were measured using the standard PHQ-2 scale. Participants were asked how often over the last 2 weeks they had felt down, depressed, or hopeless, or had little interest or pleasure in doing things. Responses were 0 days, 1–7 days, 8–12 days, 13 or 14 days, or refused to answer. A scale of 0–6 is created based on these responses, and a cut-off of 2 was used because it has been shown to have high sensitivity in detecting depressive symptoms (28). Participants who reported depressive symptoms in the past 2 weeks preceding the survey were asked if they were experiencing it more during the pandemic than before. In this paper, we examine the proportion of participants who reported having experienced depressive symptoms more during the pandemic compared to before the pandemic.

#### Anxiety disorders

Anxiety disorders were measured using the standard GAD-2 scale. Participants who reported anxiety symptoms in the past 2 weeks preceding the survey were asked if they were experiencing it more during the pandemic than before. In this paper, we examine the proportion of participants who reported having experienced anxiety disorders more during the pandemic compared to before the pandemic.

### Covariates

We considered several explanatory variables (covariates) including socio-demographic characteristics (age, sex, marital status, education level, and site) and the COVID-19 socio-economic effects including skipping meals, income loss, not accessing needed health services, seeing family less, and experiencing more violence in the house or the neighborhood. In this paper, we focus on the intersections of sex, age, marital status, and residence location. The variables are defined as below:

*Complete loss of income:* Self-reported measure dummy coded 1 if lost income completely due to COVID-19, 0 otherwise.

*Partial loss of income:* Self-reported measure dummy coded 1 if lost income partially due to COVID-19, 0 otherwise.

*Being unable to see friends and family as frequently as desired:* Self-reported measure dummy coded 1 if reported that they were seeing their family or friends less due to COVID-19, 0 otherwise.

*Experiencing more violence in the house or neighborhood*: Experiencing or witnessing crime or violence in the house or neighborhood coded 1, 0 otherwise.

*Not accessing needed health services:* Not accessing health care/services/medicines that you would have otherwise needed coded 1, 0 otherwise.

*Skipping meals:* Whether in the past 7 days, the participant or someone in the household had eaten less or skipped meals because they did not have enough money or food as a result of the COVID-19 situation (coded 1, 0 otherwise).

### Statistical analysis

Frequencies and proportions were used to summarize categorical variables and Chi-square tests were used to assess differences between groups. Mean and standard deviation were used to summarize numeric variables and t-tests were used to examine any differences between groups. Modified Poisson regression model was used to examine the association between COVID-19 socio-economic effects and mental health outcomes. In the regression models, a new composite binary variable was created by combining depressive symptoms and anxiety disorders—those that reported either increased depressive symptoms or increased anxiety disorders were coded 1, and otherwise 0. We included the socio-economic effects of COVID-19 and controlled for time-related variations by including the survey round as a covariate. Model diagnostics included running post-estimation tests for model specification and overdispersion. These included tests for model specification and Chi-square goodness of fit test dispersion. A significant coefficient for the model specification test and goodness of fit (GOF) test suggests model misspecification and overdispersion, respectively. An intersectionality analysis approach was used to examine any disproportionate associations between COVID-19 socio-economic effects on mental health outcomes based on sex, age, marital status, and residence location. Coefficient plots are presented to illustrate the results.

## Results

### Participant’s socio-demographic characteristics

Table 1 summarizes the socio-demographic characteristics of the study sample based on data collected in the fourth round. In February 2021, 4,050 participants were interviewed and the median age of participants from all sites was 39 years old (IQR = 29–47) with male participants being older on average [median: 42 (IQR: 32–51)] compared to female [median:37 (IQR:28–43)]. Participants from Kilifi were on average older, median age of 48 years (IQR = 42–56) among males and 40 years (IQR = 34–46) among females, than participants from the two other sites. Overall, two-thirds of the participants were female, with Kisumu having a higher representation of female participants than males. Education distribution varied by site with Nairobi and Kisumu having a higher distribution of participants with secondary education or more than Kilifi. Across all the sites, about a quarter of the female participants were separated, widowed, or divorced compared to male participants (3–5%).

**Table 1:** Participants socio-demographic characteristics based on data at round 4.

	All (*n* = 4,050)		Nairobi (*n* = 1,903)		Kilifi (*n* = 1,291)		Kisumu (*n* = 856)	
	Male (*n* = 1,353)	Female (*n* = 2,697)	Male (*n* = 707)	Female (*n* = 1,196)	Male (*n* = 508)	Female (*n* = 783)	Male (*n* = 138)	Female (*n* = 718)
Age [Median (IQR)]	42 (32–51)	37 (28–45)	38 (25–46)	36 (29–43)	48 (42–56)	40 (34–46)	42 (30–51)	34 (23–44)
Age categories in years								
18–29	304 (22.5)	725 (26.9)	240 (34.0)	305 (25.5)	31 (6.1)	96 (12.3)	33 (23.9)	324 (45.1)
30–49	653 (48.3)	1,601 (59.4)	353 (49.9)	768 (64.2)	235 (46.3)	539 (68.8)	65 (47.1)	294 (41.0)
50+	396 (29.3)	371 (13.8)	114 (16.1)	123 (10.3)	242 (47.64)	148 (18.9)	40 (29.0)	100 (13.9)
Education								
Primary or less	645 (47.8)	1,629 (60.5)	226 (32.0)	600 (50.3)	351 (69.2)	682 (87.2)	68 (49.6)	347 (48.4)
Secondary	504 (37.3)	846 (31.4)	335 (47.5)	490 (41.0)	122 (24.1)	75 (9.6)	47 (34.3)	281 (39.2)
Higher	201 (14.9)	104 (8.7)	145 (20.5)	104 (8.7)	34 (6.7)	25 (3.2)	22 (16.1)	89 (12.4)
Marital status								
Not married	200 (14.8)	551 (20.5)	177 (25.1)	294 (24.6)	14 (2.8)	77 (9.9)	9 (6.6)	180 (25.1)
Married	1,103 (81.7)	1,491 (55.4)	511 (72.4)	601 (50.3)	469 (92.5)	521 (66.9)	123 (89.8)	369 (51.5)
Separated/Divorced/Widowed	47 (3.5)	651 (24.2)	18 (2.6)	299 (25.0)	24 (4.7)	184 (23.5)	5 (3.7)	168 (23.4)

Except stated otherwise, all results are presented as *n* (%).

### COVID-19 socio-economic effects

Table 2 shows COVID-19 gendered socio-economic effects in the fourth and fifth rounds of the survey in Nairobi, Kilifi, and Kisumu. Overall, 33% (male 31%, female 34%) and 27% (male 23%, female 31%) of the participants in the two rounds completely lost their income due to COVID-19. This varied by site being highest in Nairobi at 39% (male 35%, female 42%) at round four and 32% (male 28%, female 35%) at round five followed by Kilifi and Kisumu. The overall proportion of participants who partially lost their income was 49% (male 55%, female 43%) in round four and 55% (male 62%, female 48%) in round five. We observed that the proportion of those who partially lost income due to COVID-19 was highest among participants from Kisumu and Nairobi at both rounds. More males than females partially lost income in all three sites. Social and family interactions were the most affected by COVID-19-related measures with 89% (male 88%, female 89%) of the participants in the fourth round and 74% (male 71%, female 77%) in the fifth round reporting that they were not able to see their families as often as they would have wished. Overall, this affected both males and females similarly though it was highest in Nairobi at 93% (male 92%, female 93%), followed by Kisumu at 88% (male 86%, female 90%) and Kilifi at 85% (male 85%, female 85%).

**Table 2:** COVID-19 socio-economic effects in Kilifi, Kisumu, and Nairobi.

		Total (Male) (R4 *n* = 1,226) (R5 *n* = 972)	Total (Female) (R4 *n* = 2,440) (R5 *n* = 1945)	Kilifi (Male) (R4 *n* = 506) (R5 *n* = 439)	Kilifi (Female) (R4 *n* = 781) (R5 *n* = 658)	Kisumu (Male) (R4 *n* = 137) (R5 *n* = 112)	Kisumu (Female) (R4 *n* = 718) (R5 *n* = 590)	Nairobi (Male) (R4 *n* = 583) (R5 *n* = 421)	Nairobi (Female) (R4 *n* = 942) (R5 *n* = 697)
Complete loss of income	R4	380 (31.0)	840 (34.4)	161 (31.8)	235 (30.1)	18 (13.1)	207 (28.8)	201 (34.5)	398 (42.3)
	R5	223 (22.9)	597 (30.7)	87 (19.8)	158 (24.1)	17 (15.2)	194 (32.9)	119 (28.3)	245 (35.2)
Partial loss of income	R4	675 (55.0)	1,053 (43.2)	239 (47.2)	274 (35.1)	106 (77.4)	382 (53.2)	329 (56.4)	397 (42.2)
	R5	601 (61.8)	936 (48.1)	252 (57.4)	303 (46.1)	87 (77.7)	279 (47.3)	262 (62.2)	354 (50.8)
See family or friends less	R4	1964 (88.4)	2,181 (89.4)	429 (84.8)	661 (84.6)	118 (86.1)	644 (89.7)	537 (92.1)	876 (93.0)
	R5	693 (71.3)	1,499 (77.1)	291 (66.3)	470 (71.4)	88 (78.6)	467 (79.2)	314 (74.6)	562 (80.6)
Experience more violence in the house	R4	652 (53.2)	1,386 (56.8)	195 (38.5)	300 (38.4)	86 (62.8)	460 (64.1)	371 (63.6)	626 (66.5)
	R5	484 (49.9)	1,076 (55.3)	171 (39.0)	246 (37.4)	69 (61.6)	418 (70.9)	244 (58.1)	412 (59.1)
Did not access needed health services	R4	80 (6.5)	245 (10.1)	23 (4.6)	28 (4.9)	8 (5.8)	68 (9.5)	23 (5.6)	139 (14.8)
	R5	100 (10.3)	297 (15.3)	48 (10.9)	81 (12.3)	22 (19.6)	132 (22.4)	30 (7.1)	84 (12.1)
Skipped meals	R4	762 (62.2)	1,633 (66.9)	279 (55.1)	427 (54.7)	84 (61.3)	507 (70.6)	399 (68.4)	699 (74.2)
	R5	496 (51.0)	1,143 (58.8)	200 (45.5)	341 (51.8)	76 (67.9)	402 (68.1)	220 (52.3)	400 (57.4)

R4, Round 4 (July 2020); R5, Round 5 (February 2021); All results are presented as *n* (%); Except stated otherwise, all results are presented as *n* (%).

Slightly more females than males experienced more violence in the house during COVID-19. Over half of the participants (male 53%, female 57%) in round four and 53% (male 50%, female 55%) in round five experienced more violence in the house. By site, Kisumu had the highest proportion at 64% (male 63%, female 64%) in round four and 67% (male 62%, female 71%) in round five, and Nairobi at 65% (male 64%, female 67%) in round four and 59% (male 58%, female 59%) in round five. Access to health care is important during a pandemic but 9% (male 7%, female 10%) and 13% (male 10%, female 15%) of the participants reported that they were not able to access the needed essential healthcare services in rounds four and five, respectively. The proportion of participants who did not access needed healthcare services was highest in Kisumu during the fifth round at 21% (male 20%, female 22%) compared to Nairobi at 10% (male 7%, female 12%) and Kilifi at 12% (male 11%, female 12%). Another important factor during a pandemic is food security. Close to two-thirds (male 62%, female 67%) and over half of the participants (male 51%, female 59%) of the participants in rounds four and five, respectively, reported that they had skipped a meal because of lack of sufficient food in the household. This effect was more pronounced in Nairobi in the fourth round at 72% (male 68%, female 74%) followed by Kisumu at 66% (male 61%, female 71%) and Kilifi at 49% (male, 46%, female 52%). Overall, on average, the effect was slightly higher among females than males.

### Mental health outcomes by gender and site

The proportions of participants reporting depressive symptoms and anxiety disorders disaggregated by sex and survey round are given in Table 3. In general, we observe higher proportions of anxiety disorders and depressive disorders among females than in males across all study sites in the fifth round except Kilifi, where more males experienced depressive and anxiety disorders than females.

**Table 3:** Mental health outcomes by gender and site.

		Depressive symptoms				Anxiety disorders			
		All	Male	Female	*p*-value	All	Male	Female	*p*-value
Overall	R4	2,404 (59.4)	796 (58.8)	1,608 (59.6)	0.63	–	–	–	–
	R5	1,502 (40.8)	451 (36.6)	1,051 (43.0)	<0.01	1,306 (35.5)	377 (30.6)	929 (38.0)	<0.01
Kilifi	R4	733 (56.8)	303 (59.6)	430 (54.9)	0.09	–	–	–	–
	R5	403 (31.4)	173 (34.3)	230 (29.5)	0.07	314 (24.4)	133 (26.3)	181 (23.2)	0.20
Kisumu	R4	624 (72.9)	101 (73.2)	523 (72.8)	0.93	–	–	–	–
	R5	451 (52.9)	67 (48.9)	384 (53.7)	0.30	383 (45.0)	56 (40.9)	327 (45.7)	0.29
Nairobi	R4	1,047 (55.0)	392 (55.4)	655 (54.8)	0.77	–	–	–	–
	R5	648 (42.0)	211 (35.7)	437 (45.9)	<0.01	609 (39.5)	188 (31.8)	421 (44.2)	<0.01

Dash (−) means data were not collected for that round; R4, Round 4 (July 2020); R5, Round 5 (February 2021); Except stated otherwise, all results are presented as n (%).

COVID-19 socio-economic effects on mental health outcomes based on the intersection of gender, age, and marital status.

The different intersections of key socio-demographic variables (gender, age, and marital status) were further examined on how COVID-19 socio-economic effects were associated with their overall mental health condition. These are presented in the form of coefficient plots and adjusted prevalence ratios (aPR) Detailed results of the regression are shown in Table 4.

**Table 4:** Modified poisson regression on the gendered effects of COVID-19 on mental health.

(a) Overall						
	Male	Female	–	–	–	–
	aPR (95% CI)	aPR (95% CI)	–	–	–	–
Complete loss of income	1.22 (1.00, 1.48)*	1.16 (1.03, 1.30)*	–	–	–	–
Partial loss of income	1.02 (0.85, 1.22)	1.09 (0.97, 1.22)	–	–	–	–
See family/friends less	1.18 (1.00, 1.38)*	1.06 (0.94, 1.18)	–	–	–	–
Experienced more violence in the house	1.30 (1.15, 1.47)**	1.17 (1.07, 1.27)**	–	–	–	–
Did not access necessary health services	0.96 (0.79, 1.17)	1.11 (1.00, 1.24)	–	–	–	–
Skipped meals	1.46 (1.29, 1.65)**	1.38 (1.26, 1.51)**	–	–	–	–
*Site (Ref = Kilifi)*			–	–	–	–
Kisumu	1.20 (1.00, 1.44)	1.38 (1.23, 1.54)**	–	–	–	–
Nairobi	1.02 (0.89, 1.18)	1.29 (1.17, 1.44)**	–	–	–	–

(b) Gender and age intersection						
	18–29 years		30–49 years		50+ years	
	Male	Female	Male	Female	Male	Female
	aPR (95% CI)	aPR (95% CI)	aPR (95% CI)	aPR (95% CI)	aPR (95% CI)	aPR (95% CI)
Complete loss of income	1.09 (1.07, 1.11)**	1.04 (0.94, 1.14)	1.30 (0.93, 1.82)	1.19 (0.99, 1.43)	1.22 (1.17, 1.27)**	1.33 (1.17, 1.51)**
Partial loss of income	0.81 (0.76, 0.87)**	0.97 (0.89, 1.06)	1.14 (1.12, 1.16)**	1.12 (0.89, 1.41)	1.00 (0.83, 1.21)	1.25 (1.22, 1.29)**
See family/friends less	1.44 (1.30, 1.59)**	1.13 (1.08, 1.18)**	1.24 (1.07, 1.43)**	1.03 (0.98, 1.07)	1.04 (0.81, 1.35)	1.10 (0.98, 1.23)
Experienced more violence in the house	1.17 (1.02, 1.34)*	1.30 (1.17, 1.43)**	1.31 (1.30, 1.32)**	1.14 (1.04, 1.24)**	1.42 (1.30, 1.56)**	1.13 (0.99, 1.29)
Did not access necessary health services	0.89 (0.75, 1.06)	1.21 (1.05, 1.40)*	0.98 (0.89, 1.08)	1.04 (0.98, 1.11)	0.91 (0.64, 1.31)	1.15 (0.95, 1.38)
Skipped meals	1.35 (0.84, 2.17)	1.37 (1.29, 1.45)**	1.48 (1.31, 1.68)**	1.43 (1.24, 1.66)**	1.53 (1.26, 1.86)**	1.20 (0.97, 1.49)
*Site (Ref = Kilifi)*						
Kisumu	1.31 (0.94, 1.82)	1.49 (0.94, 2.35)	1.20 (1.12, 1.28)**	1.30 (0.98, 1.73)	1.14 (0.89, 1.47)	1.41 (1.11, 1.79)**
Nairobi	1.06 (0.75, 1.50)	1.25 (0.69, 2.23)	1.02 (0.92, 1.14)	1.29 (0.93, 1.80)	0.97 (0.91, 1.04)	1.41 (1.08, 1.84)**

(c) Gender intersection and marital status intersection						
	Not married		Married		Sep/Divorced/Wid	
	Male	Female	Male	Female	Male	Female
	aPR (95% CI)	aPR (95% CI)	aPR (95% CI)	aPR (95% CI)	aPR (95% CI)	aPR (95% CI)
Complete loss of income	1.06 (0.94, 1.19)	1.08 (1.02, 1.14)*	1.25 (1.12, 1.38)**	1.20 (1.04, 1.39)*	1.00 (0.55, 1.82)	1.13 (1.01, 1.27)*
Partial loss of income	0.77 (0.73, 0.80)**	1.01 (0.93, 1.10)	1.07 (0.92, 1.24)	1.11 (0.93, 1.33)	0.64 (0.56, 0.74)**	1.10 (1.03, 1.17)**
See family/friends less	1.92 (1.55, 2.37)**	1.15 (1.13, 1.17)**	1.11 (0.89, 1.39)	1.06 (0.99, 1.14)	1.86 (0.97, 3.56)	1.01 (0.95, 1.08)
Experienced more violence in the house	1.25 (0.94, 1.66)	1.28 (1.16, 1.42)**	1.32 (1.25, 1.39)**	1.15 (0.99, 1.34)	1.10 (0.92, 1.31)	1.15 (1.11, 1.20)**
Did not access necessary health services	0.94 (0.82, 1.08)	1.16 (1.11, 1.21)**	0.97 (0.78, 1.19)	1.08 (1.07, 1.09)**	0.65 (0.34, 1.25)	1.12 (0.98, 1.28)
Skipped meals	1.39 (0.86, 2.23)	1.37 (1.21, 1.55)**	1.49 (1.22, 1.83)**	1.42 (1.26, 1.60)**	1.01 (0.99, 1.02)	1.33 (1.23, 1.43)**
*Site (Ref = Kilifi)*						
Kisumu	1.24 (1.22, 1.27)**	1.51 (1.40, 1.62)**	1.19 (1.03, 1.38)	1.32 (0.93, 1.86)	0.97 (0.92, 1.01)	1.38 (1.05, 1.82)*
Nairobi	0.91 (0.80, 1.02)	1.17 (0.93, 1.48)	1.04 (0.92, 1.17)	1.29 (0.91, 1.84)	0.70 (0.69, 0.71)**	1.38 (0.96, 1.98)

aPR, Adjusted prevalence ratios; CI, Confidence interval; other variables controlled for in the model is education level. Model (a) adjusts for age, marital status, and education level; model (b) adjusts for marital status, age, and education level; model (c) adjusts for age and education level; * means *p* < 0.05, ** means *p* < 0.01.

### Gender disaggregated effects

**Figure 1:**
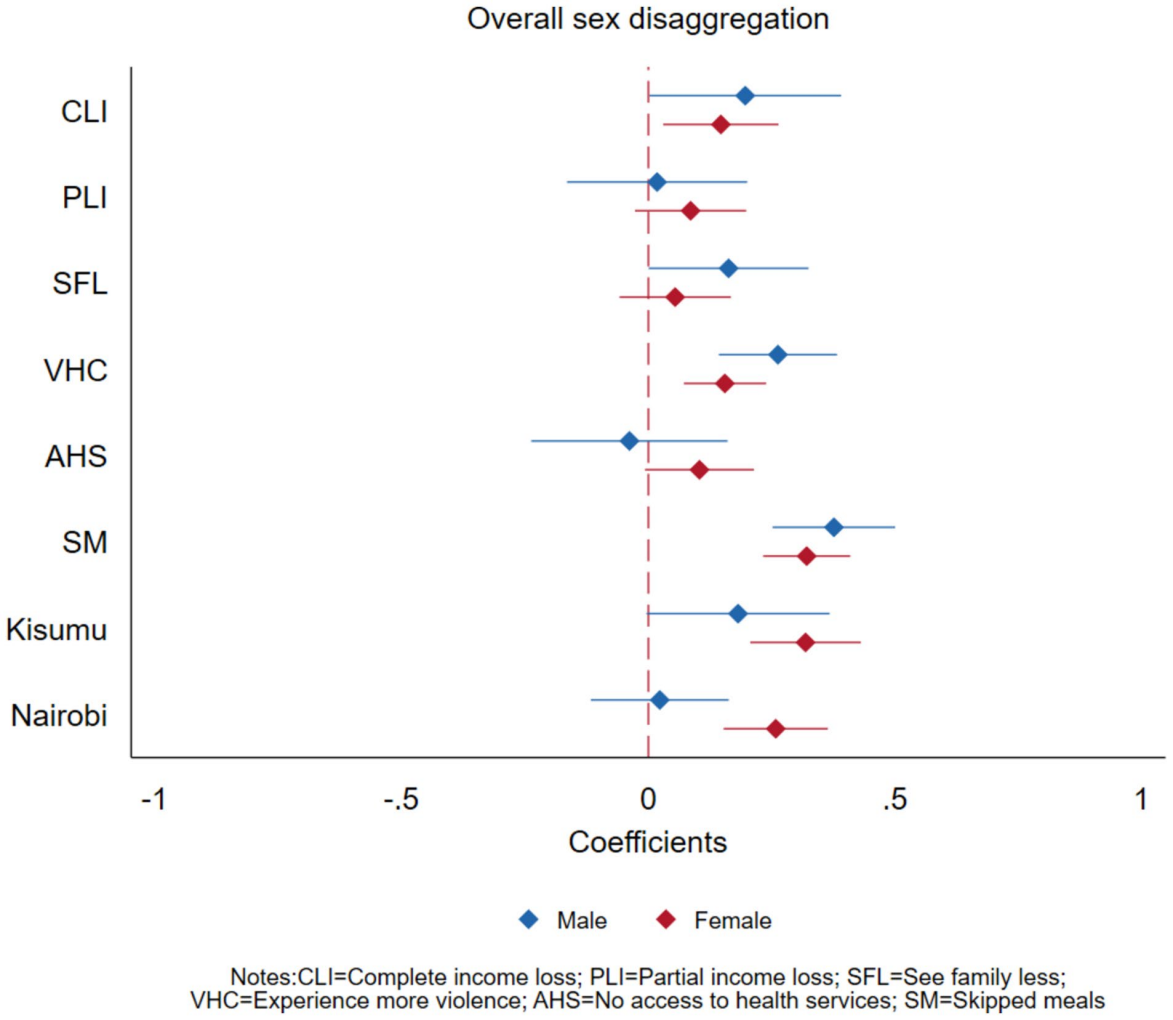
Effects of COVID-19 on mental health outcomes of adults in Nairobi, Kisumu, and Kilifi, Kenya, disaggregated by gender.

Results from Figure 1 and Table 4a show that complete loss of income was significantly associated with experiencing increased depressive symptoms or anxiety disorders for both genders but the association was stronger among the male participants (PR = 1.22, 95% CI = 1.00–1.48, *p* = 0.047) compared to the female (PR = 1.16, 95% CI = 1.03–1.30, *p* = 0.014). Partial loss of income did not have a significant association with experiencing increased depressive symptoms or anxiety disorders among both male and female participants. Access to needed health services was not significantly associated with experiencing increased depressive symptoms or anxiety disorders for both males and females at the aggregate level. Skipping meals was significantly associated with both male and female participants’ mental health at a similar magnitude though it was only significant among the females (PR = 1.37, 95% CI = 1.21–1.55, *p* < 0.001). Experiencing more violence in the house was significantly associated with experiencing increased depressive symptoms or anxiety disorders and was more pronounced among the males (PR = 1.30, 95% CI = 1.15–1.47, *p* < 0.001) compared to among the females (PR = 1.17, 95% CI = 1.07–1.27, *p* < 0.001).

### Gender and age intersection

**Figure 2:**
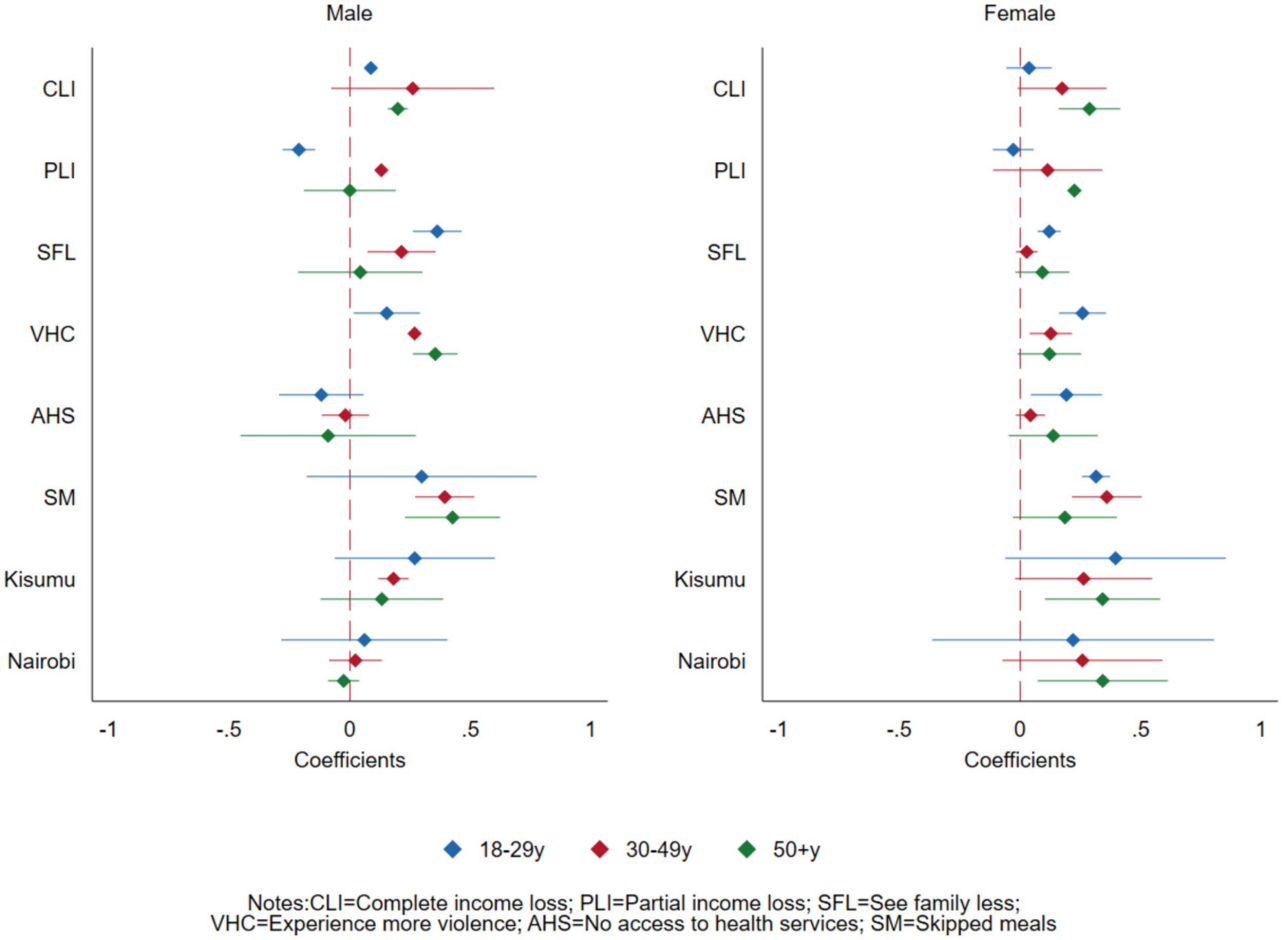
Effects of COVID-19 on mental health outcomes of adults in Nairobi, Kisumu, and Kilifi, Kenya, at the intersection of sex and age.

Figure 2 and Table 4b present findings on the gender and age intersectionality with the COVID-19 socio-economic effects on experiencing increased depressive symptoms and anxiety disorders. Complete loss of income and partial loss of income had a stronger association with experiencing increased depressive or anxiety disorder symptoms for the older (50 years or older) female participants (PR = 1.33, 95% CI = 1.17–1.51) compared to the younger females (PR = 1.04, 95% CI = 0.94–1.14). This relationship was different for younger male participants (18–29 years) where partial loss of income was negatively associated with mental health outcomes (PR = 0.81, 95% CI = 0.76–0.87).

Seeing family or friends less was more significantly associated with experiencing increased depressive or anxiety disorders for the male participants aged below 50 years but it was not significant among participants aged 50 years or older. The association between experiencing violence in the house and experiencing increased depressive or anxiety disorders increased by age among the male participants (PR: 1.17 to 1.31 to 1.42) but decreased by age among female participants (PR:1.30 to 1.14 to 1.13). Access to the needed health services was not significantly associated with experiencing increased depressive or anxiety disorders among the male participants across all the age categories but it was significant, particularly among the younger female participants (18–29 years old) (PR = 1.21, 95% CI = 1.05–1.40).

Skipping meals was significantly associated with experiencing increased depressive symptoms or anxiety disorders among the male participants across all age groups and was slightly more pronounced among the older male participants. On the other hand, while skipping meals was significantly associated with experiencing increased depression and anxiety disorders among all female age groups, it was more pronounced among the younger female participants.

There were also gender and age differences by site. Across all age groups, compared to their male counterparts, female participants from Kisumu (peri-urban) and female participants aged 50 years or older from Nairobi (PR = 1.41, 95% CI = 1.08–1.84) experienced increased depressive symptoms or anxiety disorders compared to those from Kilifi (rural).

### Gender and marital status intersection

**Figure 3:**
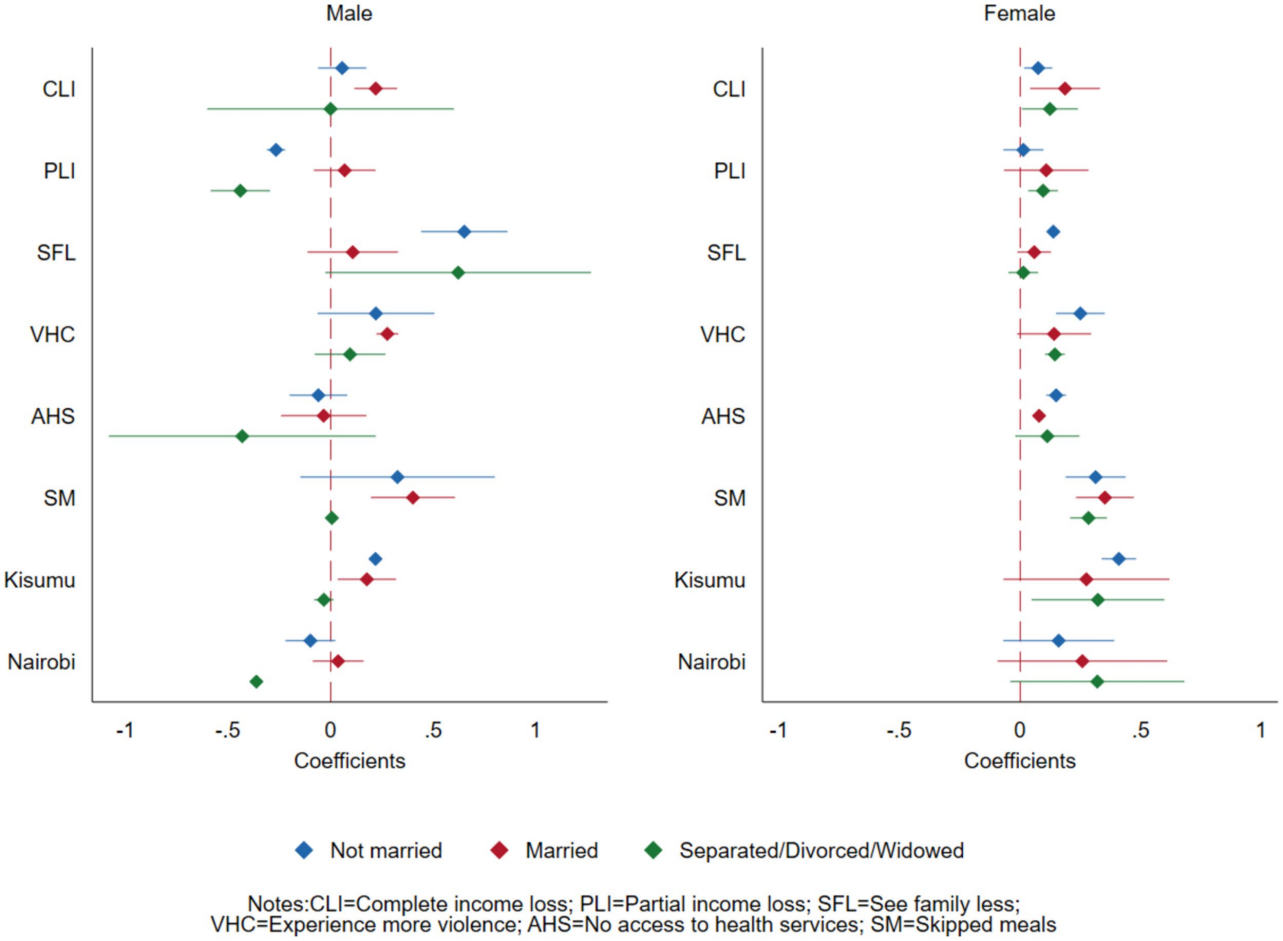
Effects of COVID-19 on mental health outcomes of adults in Nairobi, Kisumu, and Kilifi, Kenya, at the intersection of sex and marital status.

Figure 3 and Table 4c present findings on the effects of COVID-19 on experiencing increased depressive symptoms and anxiety disorders at the intersection of sex and marital status. Complete loss of income was significantly associated with experiencing increased depressive symptoms and anxiety disorders only among the married group for male participants (PR = 1.25, 95% CI = 1.12–1.38) and among all female participants (PR = 1.08 95% CI = 1.02–1.14 for not married, PR = 1.20, 95% CI = 1.04–1.39 for married and PR = 1.13, 95% CI = 1.01–1.27 among those separated, divorced or widowed).

Partial loss of income was protective against experiencing increased depressive symptoms and anxiety disorders among the male participants who were not married (PR = 0.77, 95% CI = 0.73–0.80) and males who were separated, widowed, or divorced (PR = 0.64, 95% CI = 0.56–0.74). However, it was positively associated with associated with experiencing increased depressive symptoms and anxiety disorders in females who were separated or divorced (PR = 1.10, 95% CI = 1.03–1.17).

Seeing family less was associated with experiencing increased depressive symptoms or anxiety disorders among the unmarried male participants (PR = 1.92, 95% CI = 1.55–2.37) but was not significant among the married or separated, widowed, or divorced male participants. Among female participants, it was only associated with those who were not married (PR = 1.15, 95% CI = 1.13–1.17). For both males and females, the association between skipping meals and experiencing increased depressive symptoms or anxiety disorders was more pronounced among the married (PR = 1.49, 95% CI = 1.22–1.83 male, PR = 1.42, 95% CI = 1.42–1.60 female), followed by the unmarried participants (PR = 1.39, 95% CI = 0.86–2.23 male, PR = 1.37, 95% CI = 1.21–1.55 female). Across all marital status categories, compared to their male counterparts, female participants from Nairobi (urban) and Kisumu (peri-urban) had increased depressive symptoms or anxiety disorders compared to those from Kilifi (rural).

## Discussion

In this paper, we aimed to investigate the gendered effect of COVID-19 on the mental health of adults in Nairobi, Kilifi, and Kisumu, specifically focusing on depressive and anxiety disorders. Additionally, we examined how socio-demographic characteristics (gender, age, and marital status) intersect to influence mental health outcomes among individuals in selected counties in Kenya.

We observed a significant association between economic factors and depression and anxiety outcomes. Specifically, individuals experiencing complete loss of income exhibited higher prevalence ratios for depressive and anxiety disorders, indicating a detrimental effect on their overall mental well-being. These findings resonate with previous research conducted by Salameh et al. (29) which also highlighted the substantial influence of economic instability on mental health outcomes. According to Salameh et al. the complex interplay between socio-demographic factors, pandemic-related fears, and financial well-being heightened an individual’s mental health during the pandemic. By linking the results from our study to that done by Salameh (29), we reinforce the understanding of the widespread impact of financial hardships on psychological well-being, especially during times of crisis such as the COVID-19 pandemic. This alignment underscores the importance of addressing economic disparities and implementing supportive interventions to mitigate the adverse effects on mental health. Moreover, it emphasizes the universality of these challenges across different contexts, thereby emphasizing the need for comprehensive and inclusive mental health policies and interventions.

We also noted that the direction of the association between partial loss of income and mental health outcomes was different from that of complete loss of income for different age categories of males and females. Partial loss of income was protective against negative mental health outcomes among young males (18–29 years old) but was linked to negative mental health outcomes among males aged (30–49 years old) and older females (50 years or older). This could be explained by cultural norms and societal expectations for males to be the providers for the households. This means that in times of crisis, complete loss of income should be prevented and that when designing interventions, efforts should be on protecting people from completely losing their income.

The observed variations in the prevalence of anxiety disorders across different marital statuses present compelling insights into the interplay between relationship status and mental health outcomes. Our analysis reveals that individuals who are separated, divorced, or widowed exhibit significantly higher prevalence ratios for anxiety and depressive disorders compared to their married counterparts. This finding underscores the profound impact of marital disruption on mental well-being. This result aligns closely with existing literature that has documented the association between marital dissolution and heightened psychological distress, including symptoms of anxiety and depressive disorders. A study conducted by Deegan and Dunne (30) explored the psychological ramifications of marital breakdown, highlighting factors such as loss of social support, financial strain, and disrupted family dynamics as key contributors to increased anxiety and depression levels among individuals experiencing marital dissolution. By corroborating our findings with this previous research, we provide further validation to the notion that marital disruption constitutes a significant risk factor for anxiety and depressive disorders. The body of evidence suggesting a strong link between marital status and mental health outcomes underscores the importance of considering relationship dynamics in mental health assessment and intervention strategies. Overall, our study contributes to a deeper understanding of the complex interplay between social relationships and psychological well-being.

The significance of access to mental health services as a determinant of depression and anxiety disorder outcomes cannot be overstated. Our analysis highlighted that individuals reporting unmet healthcare needs exhibited higher prevalence ratios for depressive symptoms and anxiety disorders. This underscores the pervasive barriers hindering access to mental healthcare for many individuals, encompassing financial constraints, inadequate healthcare infrastructure, and the pervasive stigma associated with mental illness. Our findings are similar to those from previous research (25, 31), which found that social support, health locus of control, and COVID-19 preventive behaviors are positively correlated with mental well-being. Our study underscores the existing disparities in mental healthcare access and emphasizes the need for policy interventions aimed at addressing these systemic challenges. Efforts should be directed toward enhancing the affordability, availability, and acceptability of mental health services to ensure equitable access for all individuals. By implementing targeted policies and initiatives, we can strive toward creating a more inclusive and accessible mental healthcare system that adequately meets the diverse needs of the population, ultimately promoting improved mental health outcomes across communities.

The analysis of gender differences in mental health outcomes across urban and peri-urban residences unveils clear disparities influenced by the intersecting dynamics of gender identity and residential context. Our findings indicate varying prevalence ratios for mental health indicators, such as depressive symptoms and anxiety disorders, among different gender groups residing in urban and peri-urban areas. In urban settings, females exhibit slightly higher prevalence ratios for depressive and anxiety disorders compared to males. This observation aligns with previous research by Zerbo et al. (32) highlighting the heightened vulnerability of women to mental health challenges in urban environments. According to the study, factors such as socioeconomic disparities, gender-based violence, and caregiving responsibilities contributed to the elevated prevalence of depressive symptoms and anxiety disorders among urban females.

In peri-urban areas, our analysis shows that females had higher prevalence ratios for mental health issues compared to males. This finding may reflect the unique stressors and socio-cultural dynamics prevalent in peri-urban settings, where traditional gender roles and economic pressures often shape mental health outcomes differently for males and females. These findings contribute to a deeper understanding of the complex interplay between gender, urbanization, and mental health. By recognizing and addressing these intersecting dynamics, policymakers and practitioners will be able to develop targeted interventions that promote mental health equity and resilience for all gender groups, irrespective of their residential context.

Female participants who did not access essential health services reported increased depressive symptoms or anxiety disorders. In this setting, women’s role as caregivers for young children and the older adults exposed women to a higher risk that they were at a higher risk of infection and diseases. It is possible that not being able to access health services impacted their ability to seek help and receive appropriate treatment for COVID-19 nor their own reproductive or mental health support for violence experienced. Access to needed health services was particularly significant for younger females and this is more likely related to access to need for reproductive health services among women of reproductive age which may increase the risk for negative reproductive health outcomes.

In contrast to studies reporting more violence among women, overall, the association between experiencing more violence in the household and experiencing increased depressive and anxiety disorders was more pronounced among males. Typical in Africa, regardless of social or health status, every man should take responsibility for his family which is a societal expectation attached to the role of a man (33). Previous studies have shown that men are less likely to report violence than female (34). Future programs could address men’s stigma, negative societal norms, and seeking support for mental health. There is evidence that the experience of violence among women in Kenya increased during COVID-19 (35). While in environments where women face violence, the inability to report or access health services can prolong their suffering and increase the risk of developing negative mental health outcomes. Strengthening support services for victims and survivors and enhancing domestic violence prevention mechanisms should be prioritized to ensure the safety and well-being of individuals within households. The association between violence in the household and mental health outcomes increased with age among males but decreased among females. Older men are more likely to have larger families and societal responsibilities than younger men so it is likely that pressure to fend for larger families increases their risk for stress.

Unmarried males and younger males were more impacted by reduced social interactions on mental health. Access to supportive social networks and communities plays a crucial role in mental health (36). This may have been more pronounced among unmarried males and young males because the restricted movements and social interactions meant that they had to stay alone in their houses. This lack of social support can contribute to feelings of loneliness and depression. Promoting social support networks and community resilience is essential in mitigating the negative impacts of reduced social interactions and isolation. Programming interventions should support tailored initiatives such as community-based support groups, online mental health resources, and peer support programs to provide individuals or groups with coping mechanisms and social connections during challenging times.

Females from urban and peri-urban areas showed increased mental health challenges compared to those from rural areas across marital status categories. This is expected given that urban areas often have a faster-paced and more competitive lifestyle, have high demand for jobs, and may experience environmental stressors like pollution, overcrowding, and poor sanitation leading to increased stress levels among residents especially in informal settlements. Mental health challenges may have been related to the economic downturn seen during lockdowns (as most women rely on casual employment, micro- to small- enterprises, and daily wages) which they had lost.

This study has strengths. First, the survey was among the first few to be conducted in Kenya after the COVID-19 pandemic providing data on how the COVID-19 pandemic affected the socio-economic and health well-being of people in informal settlements in Kenya. Second, the present analysis uses an intersectionality approach revealing unique gender dynamics including vulnerability groups as far as COVID-19-related socio-economic impacts on the general adult population’s mental health effects are concerned.

The use of a large and diverse sample population enhances the generalizability of the findings, allowing for broader implications and applicability to similar contexts. Additionally, the comprehensive analysis of prevalence ratios offers a robust statistical framework for assessing gender disparities in mental health outcomes within distinct residential settings. The identification of gender-specific stressors and vulnerabilities in urban and peri-urban environments lays the groundwork for targeted policy interventions and support systems tailored to the diverse needs of different gender groups. In general, the strengths of this research lie in its comprehensive approach, robust methodology, and potential to inform actionable strategies for improving mental health outcomes across varied residential contexts.

Despite its strengths, this research has some limitations. The limitations are largely similar to those outlined by Jessie et al. (5) who analyzed adolescent mental health using the dataset from the same survey in the fourth round. First, our survey respondents are not representative of all adults in Kenya because we only collected data in Nairobi, Kisumu, and Kilifi where we had an existing database of participants or household contacts that could be called on a phone. Further, interviews were conducted by phone which means that some participants may have been left out or not reached if they did not have access to a phone. This proportion however is small because there is generally high phone coverage in our study settings (37). Second, data collection was conducted over the phone and therefore reliability of the responses, which were self-reported, may not be ascertained to the highest degree. During the pandemic, however, the only way to collect data was to utilize mobile phone surveys because the government put in place containment measures including lockdowns in high-risk areas, and restricting physical person-to-person interactions for non-essential services. Second, we used the PHQ-2 rather than the usual PHQ-9 tool to measure depressive symptoms, and GAD-2 rather than the robust GAD-7 to measure anxiety disorder. This was mainly because the tools had to be short to facilitate rapid data collection since the focus was on getting quick data to inform the pandemic response. Nonetheless, the PHQ-2 and GAD-2 have been shown to reliably estimate depressive symptoms in a variety of settings (38, 39).

Additionally, the reliance on self-reported data may introduce response bias and underreporting of mental health symptoms, potentially affecting the accuracy of prevalence estimates. The lack of qualitative data also limits the depth of understanding regarding the lived experiences and perceptions of mental health among individuals in urban and peri-urban areas. Future research incorporating mixed-methods approaches and longitudinal designs could address these limitations and provide a more nuanced understanding of the complex relationships between gender, residential context, and mental health outcomes. We also acknowledge the limitations of causality in our study. Our study cannot infer the size and direction of the effect on mental health attributable to COVID-19 since we did not have a control arm (counterfactual).

In conclusion, the findings from this paper highlight the intricate web of socio-demographic factors influencing the mental health impacts of the COVID-19 pandemic. Our study highlights the disproportionate effects experienced by certain subgroups of the population, particularly those residing in informal settlements. This underscores the need for targeted programs and policy interventions to improve public health responses to mental health during pandemics.

Effective policy interventions should prioritize measures aimed at mitigating the adverse effects of economic instability, ensuring access to essential health services, and addressing food insecurity. Our analysis reveals that individuals who experience a complete loss of income are particularly vulnerable to negative mental health outcomes. Therefore, interventions aimed at preventing income loss and providing economic support could serve as crucial protective factors against the psychological toll of the pandemic. Furthermore, access to mental health services emerges as a critical determinant of mental health outcomes. It is imperative to address the barriers hindering access to care, including financial constraints and stigma surrounding mental illness. Policy interventions and future research could focus on improving the affordability, availability, and acceptability of mental health services to ensure equitable access for all individuals, irrespective of socio-demographic background.

The multifaceted nature of mental health disparities requires a comprehensive and integrated approach to intervention and policy development. By addressing economic inequities, strengthening social support networks, and enhancing access to culturally competent mental health services, policymakers can effectively reduce mental health disparities and promote well-being across diverse communities, including informal settlements. By drawing upon insights from this research and previous studies, policymakers can implement evidence-based interventions that prioritize mental health equity and resilience in the face of public health crises.

## Data Availability

The datasets presented in this study can be found in online repositories. The names of the repository/repositories and accession number(s) can be found at: https://dataverse.harvard.edu/dataset.xhtml?persistentId=doi:10.7910/DVN/VO7SUO. Any other data from subsequent analysis will be deposited in this repository.
